# SDS-induced oligomerization of Lys49-phospholipase A_2_ from snake venom

**DOI:** 10.1038/s41598-019-38861-8

**Published:** 2019-02-20

**Authors:** Takashi Matsui, Shizuka Kamata, Kentaro Ishii, Takahiro Maruno, Nouran Ghanem, Susumu Uchiyama, Koichi Kato, Atsuo Suzuki, Naoko Oda-Ueda, Tomohisa Ogawa, Yoshikazu Tanaka

**Affiliations:** 1 Graduate School of Life Sciences, Tohoku University, 2-1-1 Katahira, Aoba-ku, Sendai, Miyagi 980-8577 Japan; 2 Exploratory Research Center on Life and Living Systems (ExCELLS), National Institutes of Natural Sciences, 5-1 Higashiyama, Myodaiji-cho, Okazaki, 444-8787 Japan; 3 Graduate School of Engineering, Osaka University, 2-1 Yamadaoka, Suita, Osaka, 565-0871 Japan; 4 Graduate School of Pharmaceutical Sciences, Nagoya City University, 3-1 Tanabe-dori, Mizuho-ku, Nagoya, 467-8603 Japan; 5 Institute for Molecular Science, National Institutes of Natural Sciences, 5-1 Higashiyama, Myodaiji-cho, Okazaki, 444-8787 Japan; 6 Department of Biomolecular Engineering, Graduate School of Engineering, Nagoya University, Furo-cho, Chikusa-ku, Nagoya, 464-8603 Japan; 7 Faculty of Pharmaceutical Sciences, Sojo University, 4-22-1 Ikeda, Nishi-ku, Kumamoto, 860-0082 Japan; 8 Japan Science and Technology Agency, PRESTO, 2-1-1 Katahira, Aoba-ku, Sendai, 980-8577 Japan

## Abstract

Phospholipase A_2_ (PLA_2_) is one of the representative toxic components of snake venom. PLA_2_s are categorized into several subgroups according to the amino acid at position 49, which comprises either Asp49, Lys49, Arg49 or Ser49. Previous studies suggested that the Lys49-PLA_2_ assembles into an extremely stable dimer. Although the behavior on Sodium dodecyl sulfate-polyacrylamide gel electrophoresis (SDS-PAGE) under reducing or non-reducing conditions suggested the presence of intermolecular disulfide bonds, these bonds were not observed in the crystal structure of Lys49-PLA_2_. The reason for this discrepancy between the crystal structure and SDS-PAGE of Lys49-PLA_2_ remains unknown. In this study, we analyzed a Lys49-PLA_2_ homologue from *Protobothrops flavoviridis* (*Pfl*Lys49-PLA_2_ BPII), by biophysical analyses including X-ray crystallography, SDS-PAGE, native-mass spectrometry, and analytical ultracentrifugation. The results demonstrated that *Pfl*Lys49-PLA_2_ BPII spontaneously oligomerized in the presence of SDS, which is one of the strongest protein denaturants.

## Introduction

Snakebite induces acute myonecrosis as well as other biological effects including hemolytic, neurotoxic, cardiotoxic, anticoagulant and antiplatelet activities of multiple complex protein assemblies. These protein complexes are composed of various multi-locus gene families that underwent accelerated evolution^1^. Phospholipase A_2_ (PLA_2_, EC 3.1.1.4) is one of the representative toxic components of snake venom. Snake venom PLA_2_s are classified into groups I and II based on disulfide bond patterns. Group II PLA_2_s are further categorized into several subgroups based on the amino acid at position 49. These subgroups contain Asp49, Lys49, Arg49 or Ser49, among which Asp49 and Lys49 are the most common. Although PLA_2_s share high sequence similarity, their phospholipase activity is distinct. Asp49-PLA_2_ hydrolyzes the *sn-2* ester bond of the membrane phospholipids to generate fatty acids and lysophospholipids in a Ca^2+^ dependent manner. The Asp49 coordinating on the Ca^2+^ ion acts as a catalytic residue for hydrolysis of the ester bond^2,3^. In contrast, Lys49-PLA_2_ lacks phospholipase activity, or exhibits minimal phospholipase activity because the catalytic Asp49 is substituted for Lys^4–6^. Despite the absence of phospholipase activity, Lys49-PLA_2_ exhibits myonecrotic activity due to the intrinsic function of its C-terminal region that is abundant in positive and hydrophobic residues^7–9^ which mediate caspase-independent apoptosis^10^.

Previous studies showed that the Lys49-PLA_2_ forms an extremely stable dimer which is not dissociated to monomers even in the presence of 0.1% (w/v) sodium dodecyl sulfate (SDS) and 2 M urea^11^. Even though each monomer shares a similar structure^12^, X-ray crystallography studies^4,5,7,9,13–22^ revealed two distinct dimer types: the conventional dimer and the alternative dimer. These dimers differ in their myotoxicity activity: the conventional dimer comprises the inactive state and the alternative dimer comprises the active form^12^. In the conventional dimer, two protomers interact via their β-wings and N-terminal α-helices. Compared with the conventional dimer, the alternative dimer contains a larger contact surface, generating a compact dimer conformation. The alternative dimer formation was also supported by the results of small angle X-ray scattering (SAXS)^21,23^. The gyration radius (R_g_) calculated from SAXS showed good agreement with the alternative dimer rather than the conventional dimer, suggesting that the alternative dimer has a more stable conformation in solution^21^.

Previous studies have shown that when Lys49-PLA_2_ is subjected to non-reducing SDS-polyacrylamide gel electrophoresis (PAGE), a band is generated at the position corresponding to the oligomer, and the band shifts to the position of monomer in the presence of dithiothreitol^11^. These observations suggest that Lys49-PLA_2_ contains intermolecular disulfide bonds. However, intermolecular disulfide bonds were not observed in any solved crystal structure of Lys49-PLA_2_. The reason for this discrepancy between the crystal structure and SDS-PAGE of Lys49-PLA_2_ remains unknown. Clarification of these inconsistent results may provide insight into the physiological function of this protein.

In this study, we comprehensively analyzed basic protein II (BPII), which is one of the Lys49-PLA_2_ homologues isolated from the venom of *Protobothrops flavoviridis* (*Pfl*Lys49-PLA_2_ BPII)^6,24,25^. Our biophysical analyses included X-ray crystallography, SDS-PAGE, native-mass spectrometry (Native-MS), and analytical ultracentrifugation. The results demonstrated that *Pfl*Lys49-PLA_2_ BPII spontaneously oligomerized in the presence of SDS.

## Materials and Methods

### Purification of *Pf*lLys49-PLA_2_ BPII

*P. flavoviridis* was collected at Amami Oshima Island, Kagoshima prefecture in Japan in accordance with Japanese guidelines and regulations under the law of humane treatment and management of animals as a dangerous animal. Collecting venoms was conducted after carbonic anesthesia or giving an electric shock for habu snake according to the experimental plan authorized by the Institute of Medical Science, the University of Tokyo. By this method, the pain of habu can be alleviated and accidental bite can be prevented. Snake venom was extracted from *P. flavoviridis* and was frozen quickly under liquid nitrogen and then lyophilized. The lyophilized venom was dissolved in Milli-Q water and was loaded onto a CM52 cation exchange column (15 mm i.d. x 870 mm) pre-equilibrated with 20 mM ammonium acetate buffer pH 6.8 containing 0.1 mM CaCl_2_. The bound *Pfl*Lys49-PLA_2_ BPII (UniProt ID: P0DJJ9)^6,24,25^ was eluted with a linear gradient of 20–500 mM ammonium acetate. The fractions containing *Pfl*Lys49-PLA_2_ BPII were collected and dialyzed against Milli-Q water three times utilizing a cellulose membrane (MWCO 10 kDa). The dialyzed sample was subsequently lyophilized, and stored until use.

### Sodium dodecyl sulfate-polyacrylamide gel electrophoresis (SDS-PAGE) analysis

The purity and electrophoretic characteristics of *Pfl*Lys49-PLA_2_ BPII were analyzed by 15% (w/v) SDS-PAGE followed by Coomassie Blue staining. In order to confirm the presence of intermolecular disulfide bonds, the object protein was mixed with SDS-PAGE sample buffer [62.5 mM Tris-HCl pH 6.8, 2% (w/v) SDS and 5% (w/v) sucrose] with or without 5% (v/v) 2-mercaptoethanol (2-ME). To assess the conformation of the oligomerization states in the crystal, the protein crystals were picked up from the drop, and washed three times with crystallization buffer. Next, the crystals were dissolved in SDS-PAGE sample buffer without 2-ME. All samples were incubated at 95 °C for 5 min and loaded onto the SDS-PAGE gel.

### Crystallization and X-ray crystallography

The concentration of the lyophilized protein was adjusted to 20 mg/ml with Milli-Q water. All crystallization attempts were performed using the sitting-drop vapor diffusion technique at 20 °C. Diffraction-quality crystals of the object protein were obtained with the optimized reservoir conditions [100 mM sodium acetate pH 4.2, 500 mM ammonium acetate, and 32.5% (w/v) PEG4000] after initial screenings using 96-condition crystallization screening kits (Qiagen, Hilden, Germany). All crystallization drops were prepared by mixing 1.0 µl of *Pfl*Lys49-PLA_2_ BPII with an equal volume of reservoir solution followed by equilibration of the mixtures against 50 µl of reservoir solution. The *Pfl*Lys49-PLA_2_ BPII crystals were added to a crystallization solution containing 20% (v/v) ethylene glycol as a cryoprotectant. After a few seconds, the crystals were picked up in a nylon loop and then flash-cooled to 100 K in a nitrogen gas stream. The X-ray diffraction experiments were performed at the Photon Factory (proposals 16G092 and 17G595) and SPring-8 (proposals 2015B6524, 2016A2565 and 2016B2565). X-ray diffraction data sets were collected on the BL-17A beamline at the Photon Factory (Tsukuba, Japan). The diffraction data of the crystals were processed and scaled with XDS^26^. The molecular replacements were performed with BsSP-7 (PDB code 5VFH)^16^ as the search model using Phaser^27^. The structure refinement of *Pfl*Lys49-PLA_2_ BPII was performed using phenix.refine^28^, with the twin operators of (h, -h-k, –l) with a fraction of 0.49. The structure was modified manually with COOT^29^. The quality of the final models was assessed with MolProbity^30^. All crystallographic figures were prepared with PyMOL^31^. The crystallographic data and refinement statistics are summarized in Table 1. The coordinate and structure factor data of *Pfl*Lys49-PLA_2_ BPII reported in this paper have been deposited under the accession number 6AL3.

**Table 1:** Data collection and refinement statistics.

Sample name	Lys49-PLA_2_ BPII
PDB entry	6AL3
**Data collection**	
Beamline	PF BL-17A
Wavelength (Å)	0.98000
Space group	*P*6_4_
**Unit cell parameters**	
a, b, c (Å)	126.9, 126.9, 64.9
α, β, γ (°)	90, 90, 120
Resolution range (Å)	50.0–2.57 (2.72–2.57)
Completeness (%)	99.9 (99.8)
<I/σ(I)>	12.6 (3.0)
*R*_marge_ (%)	12.3 (56.4)
CC/2	100.0 (84.6)
Multiplicity	6.7 (6.9)
No. of observed reflections	129,632 (21,169)
No. of unique reflections	19,245 (3,080)
**Refinement**	
Resolution (Å)	45.4–2.57
*R*_work_ (%)	21.8
*R*_free_ (%)	24.5
Twin fraction (%)	0.49 (*h*, -*h*-*k*, -*l*)
**r.m.s.d. from ideal**	
bond length (Å)	0.006
bond angle (°)	0.954
No. of molecules per asymmetric unit	4
**Ramachandran plot**	
favored region	91.9
allowed region	7.9
outlier region	0.2
Total atoms	3,835
Average B-factor (Å^2^)	43.0

### Native-MS analysis

Native-MS analysis was performed as described previously^32^. Briefly, 50 µM of BPII aqueous solution was measured by nanoflow electrospray ionization mass spectrometry using gold-coated glass capillaries made in house with approximately 2–5 µL of sample loaded per analysis. The spectra were recorded on a SYNAPT G2-Si HDMS mass spectrometer (Waters, Milford, Massachusetts, USA) in positive ionization mode at 1.33 kV with a 150 V sampling cone voltage and source offset voltage, 0 V trap and transfer collision energy, and 5 mL/min trap gas flow. The spectra were calibrated using 1 mg/mL cesium iodide and analyzed using MassLynx software (Waters).

### Sedimentation velocity analytical ultracentrifugation (SV-AUC)

SV-AUC experiments were conducted with 1 mg/mL of *Pfl*Lys49-PLA_2_ BPII in the presence or absence of 1% (w/v) SDS. Data collection was performed at 20 °C in a ProteomeLab XL-I analytical ultracentrifuge (Beckman Coulter, Brea, California, USA) at 60,000 rpm using UV detection. The collected data were analyzed using the continuous *c*(*s*) distribution from the program SEDFIT (version 15.01b)^33^ with fitting for the frictional ratio, meniscus, and time-invariant noise with a regularization level of 0.68. The partial specific volume of *Pfl*Lys49-PLA_2_ BPII was calculated as 0.728 cm^3^ g^−1^ using the program SEDNTERP 1.09. The buffer density and viscosity of each condition were determined using a DMA 5000 densitometer (Anton Paar, Graz, Austria) and a Lovis 2000 M viscometer (Anton Paar), respectively. The resulting data were analyzed with the program SEDNTERP 1.09. The partial specific volume of *Pfl*Lys49-PLA_2_ BPII in the presence of 1% (w/v) SDS was determined experimentally by using density contrast SV-AUC^34,35^ where the samples with identical protein concentrations but different H_2_O/D_2_O ratios (H_2_O 80%/D_2_O 20% and H_2_O 40%/D_2_O 60%) were subjected to a sedimentation velocity run. The data were analyzed by setting the partial specific volume as a floated parameter in the “Hybrid Global Continuous Distribution and Global Discrete Species” model in the program SEDPHAT (version 12.1b)^36^. The partial specific volume and molecular mass of the SDS micelle were assumed to be 0.870 cm^3^ g^−1^ and 14,000 Da, respectively^37,38^.

## Results

### Crystal structure of *Pf*lLys49-PLA_2_ BPII

**Figure 1:**
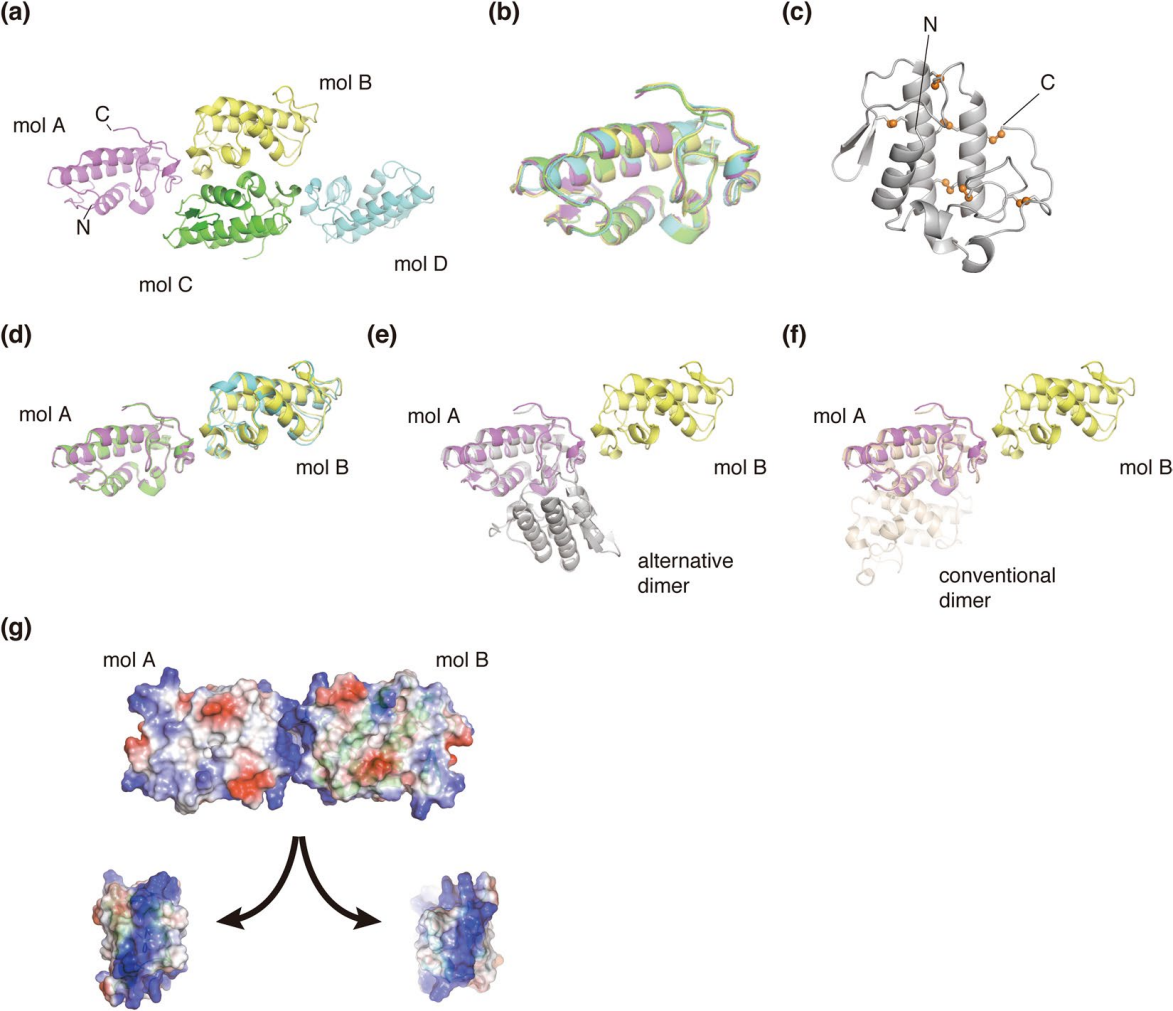
X-ray crystal structure of *Pfl*Lys49-PLA_2_ BPII. **(a**) Crystal structures of four molecules in an asymmetric unit. (**b)** Superimposition of the four molecules. **(c)** Disulfide bond network. The sulfur atoms are indicated as orange spheres. **(d)** Superimposition of molecules A and B on molecules C and D. **(e)** Superimposition of an alternative dimer (gray, PDB ID: 2Q2J) on molecule A of *Pfl*Lys49-PLA_2_ BPII. **(f)** Superimposition of the conventional dimer (wheat PDB ID: 2Q2J) on molecule A of *Pfl*Lys49-PLA_2_ BPII. **(g)** Surface charge distributions of the surface between molecules A and B. Surfaces comprising positive and negative charges are depicted as blue and red colors, respectively.

The crystal structure of *Pfl*Lys49-PLA_2_ BPII was determined at a resolution of 2.57 Å. The asymmetric unit contained four molecules of *Pfl*Lys49-PLA_2_ BPII (Fig. 1a). These four molecules were superimposed well relative to each other with root-mean-square-deviation (r.m.s.d.) values less than 0.5 Å for the Cα-atoms (Fig. 1b). All 14 Cys residues formed the following seven intramolecular disulfide bonds (Fig. 1c): Cys26–Cys116, Cys28–Cys44, Cys43–Cys96, Cys49–Cys122, Cys50–Cys89, Cys57–Cys82, and Cys75–Cys87. Ca^2+^ ions, which are important for the phospholipase activity of other Lys49-PLA_2_s was not bound on the catalytic site of *Pfl*Lys49-PLA_2_ BPII due to the substitution of Asp49 for Lys^4,5,7,13–15,17,20,22,39^.

The relative orientation between molecules A and B was similar to that between molecules C and D (Fig. 1d). The r.m.s.d. between molecules B and D after superimposing molecules A and C was 1.3 Å, which suggested the possibility that this dimer is an intrinsic dimer structure of *Pfl*Lys49-PLA_2_ BPII. However, this dimer was superimposed neither on the conventional dimer nor the alternative dimer previously reported (Fig. 1e,f). Furthermore, basic residues were located on both sides of the interface, and the surface charges of both interfaces were positive (Fig. 1g). The area of the interface among four molecules was less than 350 Å^2^. Based on these observations, we concluded that the conserved relative orientation between molecules is merely preferable packing in the crystal and *Pfl*Lys49-PLA_2_ BPII does not form any oligomers in the crystal.

### The mobility of *Pf*lLys49-PLA_2_ BPII in SDS-PAGE

**Figure 2:**
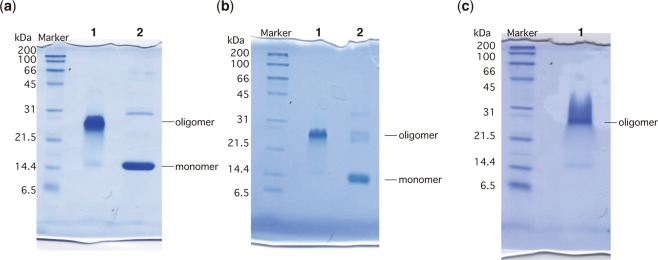
SDS-PAGE of *Pfl*Lys49-PLA_2_ BPII under various conditions. (**a)** *Pfl*Lys49-PLA_2_ BPII with and without 2-ME treatment. Lane 1, without 2-ME; lane 2, with 2-ME. **(b)** *Pfl*Lys49-PLA_2_ BPII treated with 6 M urea. Lane 1, without 95 °C treatment; lane 2, after 95 °C treatment. **(c)** Resuspension of *Pfl*Lys49-PLA_2_ BPII crystals with SDS-PAGE sample buffer.

The mobility of *Pfl*Lys49-PLA_2_ BPII was analyzed by SDS-PAGE (Fig. 2a). *Pfl*Lys49-PLA_2_ BPII without 2-ME treatment migrated at approximately 27 kDa when subjected to SDS-PAGE. On the other hand, *Pfl*Lys49-PLA2 BPII treated with 5% (v/v) 2-ME shifted to the position of approximately 14.4 kDa (Fig. 1a, lane 2). In general, the 2-ME-mediated band shift of *Pfl*Lys49-PLA_2_ BP II subjected to SDS-PAGE suggested the presence of intermolecular disulfide bond formation. However, all cysteine residues formed intramolecular disulfide bonds in the crystal structure (Fig. 1c), and thus, intermolecular disulfide bond formation is highly unlikely. These observations suggested that *Pfl*Lys49-PLA_2_ BPII may exhibit unusual behavior when subjected to SDS-PAGE. Therefore, we analyzed the mobility of *Pfl*Lys49-PLA_2_ BPII under a variety of conditions (Fig. 2b,c).

First, we evaluated the effect of urea, a typical protein denaturation reagent, on oligomerization. *Pfl*Lys49-PLA_2_ BPII was still oligomerized when subjected to SDS-PAGE in the presence of 6 M urea (Fig. 2b, lane 1). However, heat treatment in the presence of 6 M urea resulted in the dissociation of *Pfl*Lys49-PLA_2_ BPII to monomers (Fig. 2b, lane 2). It should be noted that a reducing reagent was not added to these samples. These results showed that the stable oligomer formation of *Pfl*Lys49-PLA_2_ BPII subjected to SDS-PAGE is not due to disulfide bond formation.

Next, we loaded crystals of *Pfl*Lys49-PLA_2_ BPII dissolved in the SDS-PAGE sample buffer containing 2% (w/v) SDS. Surprisingly, *Pfl*Lys49-PLA_2_ BPII, which adopted a monomeric form with no intermolecular disulfide bonds in the crystals, oligomerized after being subjected to SDS-PAGE (Fig. 2c). These observations suggested that treatment with the SDS-PAGE sample buffer induced oligomerization of this protein.

### Oligomerization state analyzed by native-MS and SV-AUC

**Figure 3:**
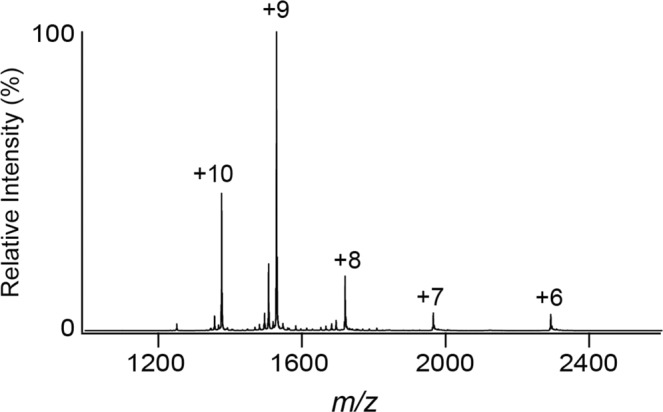
Native mass spectrum of *Pfl*Lys49-PLA_2_ BPII. Native mass spectrum of 50 µM *Pfl*Lys49-PLA_2_ BPII dissolved in water was measured under the positive ionization mode.

To identify the oligomerization state of *Pfl*Lys49-PLA_2_ BPII in an aqueous condition with neither detergent nor denaturant, the molecular mass distributions of 50 µM of the purified *Pfl*Lys49-PLA_2_ BPII dissolved in water were analyzed by native-MS. The molecular mass of *Pfl*Lys49-PLA_2_ BPII in water was determined to be 13,753 Da, which is in good agreement with the estimated molecular mass of the deduced amino acid sequence (13,872 Da) (Fig. 3). It should be noted that native-MS analysis detected no oligomer. These results revealed that *Pfl*Lys49-PLA2 BPII exists as a monomer in an aqueous solution.

**Figure 4:**
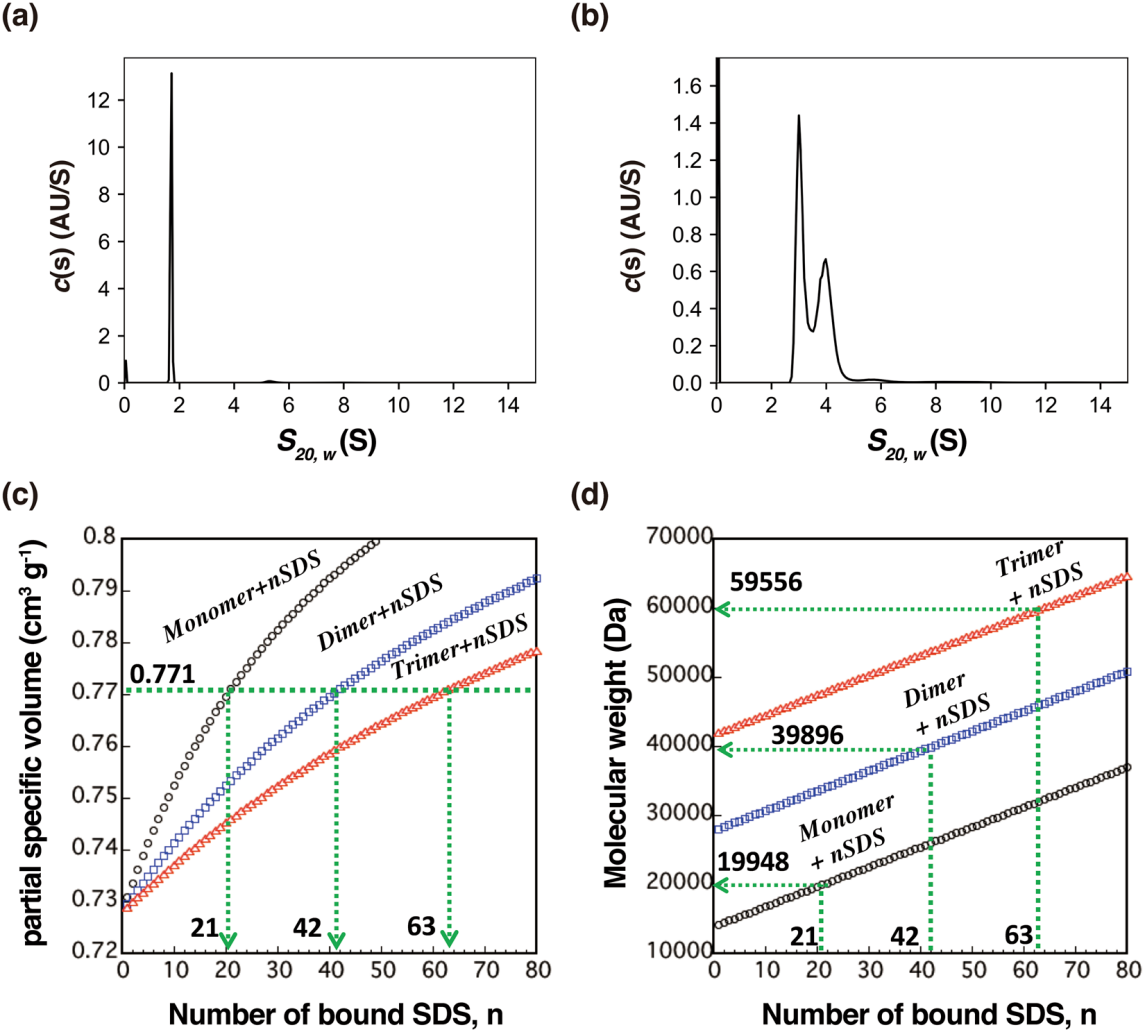
SV-AUC analysis of *Pfl*Lys49-PLA_2_ BPII. Distribution of the sedimentation coefficients of *Pfl*Lys49-PLA_2_ BPII in the absence **(a)** or presence **(b)** of 1% (w/v) SDS. The relationship between the number of bound SDS molecules and the partial specific volume **(c)** or molecular mass **(d)** of the complexes.

Next, the effect of SDS on the oligomerization state of *Pfl*Lys49-PLA_2_ BPII was analyzed by analytical ultracentrifugation. Purified *Pfl*Lys49-PLA_2_ BPII dissolved in 20 mM Tris-HCl pH 8.0 and 200 mM NaCl at a concentration of 1 mg/mL distributed as a monomer with a molecular mass of 13.1 kDa (Fig. 4a, Table 2). This result is in good agreement with the native-MS observations described above (Fig. 3). In contrast to the monomeric state of *Pfl*Lys49-PLA_2_ BPII dissolved in 20 mM Tris-HCl pH 8.0 and 200 mM NaCl, the sedimentation coefficients of *Pfl*Lys49-PLA_2_ BPII in the presence of SDS exhibited a bimodal distribution. The sedimentation coefficients significantly increased compared to those obtained in the absence of SDS (Fig. 4b).

**Table 2:** *c*(*s*) analysis of SV-AUC experiments in the presence or absence of 1% (w/v) SDS.

Condition	*s*_20,w_	% of total	*f*/*f*_0_	Estimated M_W_ (kDa)
**Without SDS**				
	1.7	95.6	1.18	13.1
	5.4	2.5		72.6
**With SDS**				
	2.6	53.9	1.24	33.1
	3.4	43.6		48.3

The density contrast SV-AUC analysis revealed that the partial specific volume of *Pfl*Lys49-PLA_2_ BPII in the presence of SDS was 0.771 cm^3^ g^−1^. Three possible protein-SDS complexes that have a partial specific volume of 0.771 cm^3^ g^−1^ are as follows: (*Pfl*Lys49-PLA_2_ BPII)(SDS)_21_, (*Pfl*Lys49-PLA_2_ BPII)_2_(SDS)_42_, and (*Pfl*Lys49-PLA_2_ BPII)_3_(SDS)_63_ of which the calculated molecular masses were 19.9 kDa, 39.9 kDa, and 59.8 kDa, respectively (Fig. 4c,d). The molecular masses obtained from *c*(*s*) analysis of the two peaks (under the assumption that the three complexes have a similar molecular shape) were 33.1 kDa for the 2.6 S peak and 48.3 kDa for the 3.4 S peak (Fig. 4b, Table 2). These results lead to the conclusion that *Pfl*Lys49-PLA_2_ BPII forms SDS bound dimers and SDS bound trimers in the presence of 1% (w/v) SDS.

## Discussion

Previous studies reported that Lys49-PLA_2_s exist as either conventional or alternative dimers, based on the results of X-ray crystallography, electrophoresis, dynamic light scattering, and spectroscopy^11,12,14,15,19,23,39–42^. In contrast to these previous reports, our present crystal structure showed that *Pfl*Lys49-PLA_2_ BPII exists as a monomer in the crystal (Fig. 1). This was consistent with the results of native-MS (Fig. 3) and analytical ultracentrifugation analyses (Fig. 4), both of which showed that monomeric *Pfl*Lys49-PLA_2_ BPII was detected in the absence of SDS. These results indicated that *Pfl*Lys49-PLA_2_ BPII exists as a monomer in both solution and crystal states.

Although the behavior of *Pfl*Lys49-PLA_2_ BPII in the crystal and solution states was distinct from Lys49-PLA_2_s from other species (i.e., *Pfl*Lys49-PLA_2_ BPII is a monomer while other Lys49-PLA_2_s assemble to dimers), the behavior upon SDS-PAGE was similar between *Pfl*Lys49-PLA_2_ BPII and other Lys49-PLA_2_s. Specifically, both proteins oligomerized upon SDS-PAGE. The dissolved crystals of *Pfl*Lys49-PLA_2_ BPII formed oligomers upon SDS-PAGE, which demonstrated that during sample preparation and/or electrophoresis the oligomerization of *Pfl*Lys49-PLA_2_ BPII occurred spontaneously (Fig. 2c). Moreover, analytical ultracentrifugation indicated that 1% (w/v) SDS induces dimerization and trimerization of *Pfl*Lys49-PLA_2_ BPII (Fig. 4). Taking these observations together, we concluded that *Pfl*Lys49-PLA_2_ BPII assembles to form SDS-resistant stable oligomers in the presence of 1% (w/v) SDS. Kilby and coworkers also reported a similar phenomenon, i.e., the interaction of SDS micelles with bovine PLA_2_ induced trimer formation, in which approximately three molecules of bovine PLA_2_ was predicted to bind one SDS micelle^43,44^. The results of our analytical ultracentrifugation showed that *Pfl*Lys49-PLA_2_ BPII assembles to form dimers and trimers. *Pfl*Lys49-PLA_2_ BPII may assemble to form oligomers in a similar manner as bovine PLA_2_. Notably, this characteristic of *Pfl*Lys49-PLA_2_ BPII in SDS-PAGE did not change even in the presence of crude snake venom (Supplementary Fig. 1), indicating no significant synergy effect from another venom components to the behavior against SDS.

It is important to note that SDS, one of the strongest protein denaturants, induces oligomerization of *Pfl*Lys49-PLA_2_ BPII. Thus, *Pfl*Lys49-PLA_2_ BPII exhibits outstandingly strong stability against chemical denaturation. Indeed, oligomers of *Pfl*Lys49-PLA_2_ BPII did not dissociate even in the presence of 6 M urea, and further heat treatment was necessary for the dissociation to monomers (Fig. 2). *Pfl*Lys49-PLA_2_ BPII harbors seven intramolecular disulfide bonds, thus 12% of the total 122 amino acids (14 Cys residues) contributes to covalent bond formation. Such a large number of intramolecular disulfide bonds may contribute to this unusual behavior of this protein, i.e., oligomerization by a protein denaturant.

In the present study, we demonstrated that SDS induces oligomerization of *Pfl*Lys49-PLA_2_ BPII. SDS can be regarded as a simple phospholipid analogue. These oligomers may also be induced by phospholipids, and the oligomer may interact with the membrane and function as a myotoxic agent to disrupt the cell membrane. Although the physiological significance of SDS-induced oligomerization of *Pfl*Lys49-PLA_2_ BPII is still unclear, it may play a key role in its myotoxic function.

## Supplementary information

Supplementary Figure 1
