# Supplementary material for: SDS-induced oligomerization of Lys49-phospholipase A2 from snake venom
Source: Sci Rep. 2019 Feb 20;9:2330. doi: 10.1038/s41598-019-38861-8 (PMC6382788; doi:10.1038/s41598-019-38861-8)
Supplement: Supplementary file 1 — Supplementary Figure 1 [file 41598_2019_38861_MOESM1_ESM.docx]

**Supplementary Material**

**SDS-induced oligomerization of Lys49-phospholipase A_2_ from snake venom**

Takashi Matsui,^1,+^ Shizuka Kamata,^1, +^ Kentaro Ishii,^2^ Takahiro Maruno,^3^ Nouran Ghanem,^1^ Susumu Uchiyama,^2, 3^ Koichi Kato,^2, 4, 5^ Atsuo Suzuki,^6^ Naoko Oda-Ueda,^7^ Tomohisa Ogawa,^1^ and Yoshikazu Tanaka^1, 8,^ *

**Supplementary Figure**


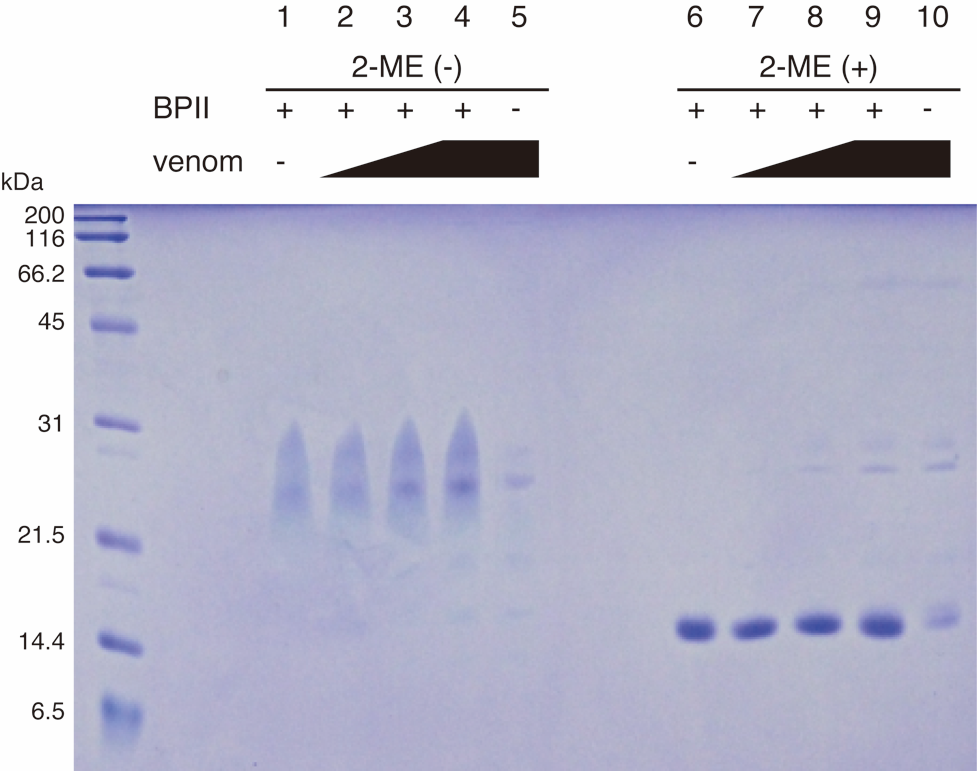


**Supplementary Figure 1.** **SDS-PAGE of *Pfl*Lys49-PLA_2_ BPII mixed with the crude snake venom.** 2.25 µg of *Pfl*Lys49-PLA_2_ BPII with (lane 6-10) and without 2-mercaptoethanol treatments (lane 1-5). Lane 1 and 6, without snake venom; lane 2-4 and 7-9, with 0.46, 1.38, 2.3 µg of snake venom; lane 5 and 10, 2.3 µg of snake venom without *Pf*Lys49-PLA_2_ BPII.
